# Genetic Analysis of Undiagnosed Juvenile GM1-Gangliosidosis by Microarray and Exome Sequencing

**DOI:** 10.1155/2018/8635698

**Published:** 2018-11-15

**Authors:** Ahmed Bouhouche, Houyam Tibar, Yamna Kriouale, Mohammed Jiddane, Imane Smaili, Naima Bouslam, Ali Benomar, Mohamed Yahyaoui, Elmostafa El Fahime

**Affiliations:** ^1^Research Team in Neurology and Neurogenetics, Genomics Center of Human Pathologies, Faculty of Medicine and Pharmacy, University Mohammed V, Rabat, Morocco; ^2^Genetics Center of the Cheikh Zayed Foundation, Abulcasis International University of Health Sciences, Rabat, Morocco; ^3^Department of Neuropediatrics and Metabolism Diseases, Hôpital d'Enfant, CHU Ibn Sina, Rabat, Morocco; ^4^Department of Neuroradiology, Hôpital des Spécialités, CHU Ibn Sina, Rabat, Morocco; ^5^Assistance Units for Scientific and Technical Research (UATRS), National Center for Scientific and Technical Research (CNRST), Rabat, Morocco

## Abstract

GM1 gangliosidosis is an autosomal recessive lysosomal storage disorder due to mutations in the lysosomal acid 3-galactosidase gene,* GLB1*. It is usually classified into three forms, infantile, juvenile, or adult, based on age at onset and severity of central nervous system involvement. Because of their broad clinical spectrum and their similarity to many other aetiologies, including inherited neurodegenerative and metabolic diseases, it is often difficult to diagnose such diseases. Recently, whole exome sequencing (WES) has become increasingly used when a strong hypothesis cannot be formulated based on the clinical phenotype. Here, we present three patients belonging to a consanguineous Moroccan family with a GM1-gangliosidosis with unusual clinical onset and atypical radiological presentation that had eluded diagnosis for over a decade. To identify the disease-causing mutation, we performed a whole exome sequencing and a chromosomal microarray genotyping in order to reduce the number of genetic variants to be interpreted, by focusing the data analysis only on the linked loci. The already known pathogenic missense mutation c.601G>A in* GLB1* (p.R201C) was found at homozygous state in the proband V.1 and at heterozygous state in his father IV.1. The mutation was validated by Sanger sequencing and segregated in all the family members according to a recessive mode of inheritance. Outside of the linked loci, we found the* EXOSC8* p.Ser272Thr mutation at heterozygous state in all the patients and their mother IV.2. This mutation was reported to cause pontocerebellar hypoplasia type 1C and could act as a modifying factor that exacerbates the brain atrophy of patients. Our study identified the first* GLB1* mutation in North Africa in patients with unexpected brain-MRI outcomes extending the clinical spectrum of the GM1-gangliosidosis.

## 1. Introduction

GM1 gangliosidosis is an autosomal recessive storage disorder, which occurs in 1 per 100,000 to 200,000 newborns [1]. It is caused by a deficiency of beta-galactosidase (GLB1), a lysosomal hydrolase that may be defective with respect to keratan sulfate in Morquio B disease (MBD) or to gangliosides, lactosylceramide, asialofetuin, and oligosaccharides carrying terminal beta-linked galactose and keratan sulfate in GM1-gangliosidosis [2]. This deficiency leads to the accumulation of keratan sulfate, glycolipids, and GM1 gangliosides in different tissues, especially in peripheral and central nervous system [3]. This disease can be divided into 3 clinical forms (type I, type II, and type III) based on the age of onset and the severity of the phenotype.

The type I or infantile form, the most severe and common [4], can start between birth and the age of 6 months, characterized by hypotonia and central nervous system degeneration. It is a rapidly progressive form as it leads to death by 1 to 2 years of age [5]. The type II is juvenile or late infantile form that starts between 7 months and 3 years of age [6, 7]. It is characterized by slower progression, delay in motor and cognitive development, muscle weakness, and seizures associated with cerebellar and extrapyramidal signs [5–8]. The type III or adult form has a late onset between 3 and 30 years and appears as extrapyramidal disorders due to local deposition of glycosphingolipid in the caudate nucleus. It may also be expressed as cerebellar dysfunctions, dystonia, slurred speech, and mild vertebral abnormalities. Skeletal changes are minor and the cherry-red spots at the macula are lacking.

The responsible gene of GM1-gangliosidosis,* GLB1*, contains 16 exons and encodes by alternative splicing to the lysosomal enzyme *β*-galactosidase and the elastin binding protein [9]. More than 110 mutations were reported as pathogenic or likely pathogenic in the GLB1 gene on the Clinvar website (ncbi.nlm.nih.gov/clinvar). These mutations, often reported at compound heterozygous state [10], give rise to proteins with a very variable enzymatic activity and to very variable clinical phenotypes [11, 12]. Because of this great clinical and genetic heterogeneity, no correlation genotype-phenotype is possible. Otherwise, other neurodegenerative diseases giving rise to a similar GM1 gangliosidosis phenotype may also complicate the diagnosis of these diseases, resulting in slow diagnosis and delay in patient management. The use of new technologies such as exome sequencing in the diagnosis of genetic diseases can help to significantly shorten these delays, avoid unproductive evaluation, and offer patients a therapeutic intervention when it is possible. In addition to including other family members in filtering variants that do not fit suspected Mendelian inheritance, prior analysis with microarray can significantly facilitate bioinformatics analysis by focusing only on the candidate loci, particularly for recessive forms.

In this study, we performed a genetic analysis in a Moroccan family displaying a neurodegenerative disease with autosomal recessive inheritance whose clinical diagnosis could not be possible for a long duration because of the clinical expression that patients presented at the onset of the disease. The genetic diagnosis of GM1 gangliosidosis due to the p.Arg201Cys mutation of the GLB1 gene in the studied family was possible and rapid by combining chromosomal microarray analysis (CMA) and whole exome sequencing (WES).

## 2. Material and Methods

### 2.1. Family Description

**Figure 1:**
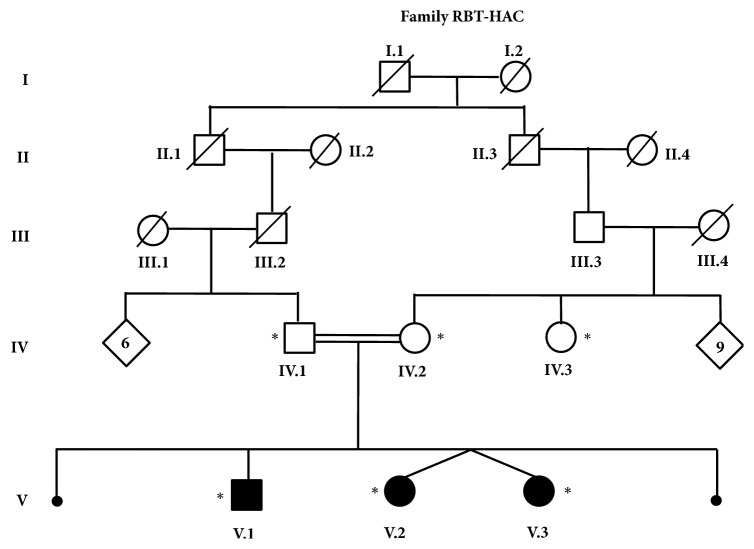
Pedigree of family RBT-HAC. Black and white symbols correspond to affected and asymptomatic members, respectively. Members who were included in the study were noticed with a star.

A consanguineous family of Moroccan origin (RBT-HAC), with two miscarriages and three affected siblings displaying an epileptic encephalopathy, was recruited in child hospital of Rabat (Morocco). The pedigree of the family is shown in Figure 1. Patients V.2 and V.3, twin sisters, were hospitalized at the child hospital and examined by neuropediatrician; whereas the oldest patient V.1 was referred to the department of neurology of Specialties Hospital of Rabat where he was examined by neurologists. Brain MRIs were obtained for all patients (V.1, V.2, and V.3) with a high field strength system (General Electric Sigma Excite2 1.5 Tesla System head coil antenna).

Blood samples were taken from patients (V.1, V.2, and V.3) and family members (IV.1, IV.2, and IV.3) and high quality genomic DNA was purified from peripheral blood leukocytes using Isolate II Genomic DNA kit from Bioline. All sampled family members or their tutors provided informed consent to participate in the study. All the studies were carried out after approval of the Moroccan ethical committee of biomedical research (CERB).

### 2.2. Microarray Genotyping and Linkage Analysis

DNA samples of the three affected children and both unaffected parents were genotyped using the CytoScan HD array from Affymetrix, which contains more than 2.6 million markers, including 750,000 genotype-able SNPs and 1.9 million nonpolymorphic probes according to the manufacturer's protocol. Briefly, 250 ng of DNA samples was digested with* Nsp1*, amplified with TITANIUM Taq DNA polymerase (Clontech, Mountain View, CA), fragmented with Affymetrix fragmentation reagent, and labeled with biotin end-labeled nucleotides. The DNA was hybridized to the microarray for 16 hours, washed and stained on the GeneChip Fluidics Station 450, and scanned on the GeneChip Scanner 3000 7G (Affymetrix). Data analysis was performed using Chromosome Analysis Suite software version 1.2.2 (Affymetrix).

The microarray data were processed using ALOHOMORA [13]. SNP data were converted to appropriate format; Graphical Representation of Relationships (GRR) program was used to evaluate familial relationship [14] whereas Mendelian errors were checked by PedCheck program [15]. The two-point and multipoint linkage analysis were performed by Merlin software program assuming an autosomal recessive mode of inheritance with complete penetrance and a disease allele frequency of 0.001.

### 2.3. Exome Sequencing

WES was performed in the patient V.3 and his father IV.1 at the Supporting Units for Technical and Scientific Research Belonging to CNRST (UATRS/CNRST, Rabat, Morocco). The target regions in the exome were amplified using Ion Proton platform (Thermo Fisher Scientific) with an Ion AmpliSeq Exome RDY Kit according to the manufacturer's protocol. It involves twelve primer pools (294 000 amplicons) which target 497% of the coding region and account for 33 Mb. All barcoded samples were sequenced on the Ion Proton with Ion PI Chips v2 taking two samples on a single chip per sequencing run. Bead templating (emulsion PCR) and chip loading were performed on Ion Chef System (Thermo Fisher Scientific). Sequence alignment to Hg19 and variant identification were performed with the Torrent Suite v.4.2.1 software. The generated VCF were then imported into the online Server of Ion Reporter Software v5.6 for variant analysis, filtering, and annotations.

### 2.4. Sanger Sequencing Validation

Sanger sequencing was used to validate the significant variants identified by exome sequencing. Briefly, exons containing the variants were amplified by PCR and both strands were sequenced using Big Dye Terminator Cycle Ready Reaction 3.1 Kits and an ABI 3130xl automated sequencer, and sequence chromatograms were analyzed using SeqScape2.1 software (Applied Biosystems, Foster City, CA).

## 3. Results

### 3.1. Clinical Finding

Patient V.1: at admission in our department, the eldest boy was 17 years old. The mother reports that the pregnancy was carried out without any problem. He walked around 1 year of age and started to talk around 2 years of age. He had a good psychomotor development until the age of 6, when he had a feverish episode that was complicated by generalized tonic-clonic seizures. These seizures were resistant to treatment. The patient then presented a progressive and significant intellectual and motor impairment, became bedridden, and lost speech. One year later, the patient started sodium valproate at 600 mg per day and Phenobarbital at 50 mg per day, and the frequency of the seizures decreased but the patient had tonic-clonic attacks complicating every febrile episode. He was addressed to our department to manage his status epilepticus, which lasted for a whole day. The clinical examination at the admission found a maintained segmental force but walking and standing were impossible, and sensitivity and coordination were difficult to explore. Positive Babinski sign was present bilaterally and osteotendinous reflexes were present in the upper limbs and difficult to explore in the lower limbs due to tendon contraction. The general examination found a thoracic deformation and tendon retractions to the four limbs. He recovered his consciousness state and was stable after adjustment of the antiepileptic treatment.

**Figure 2:**
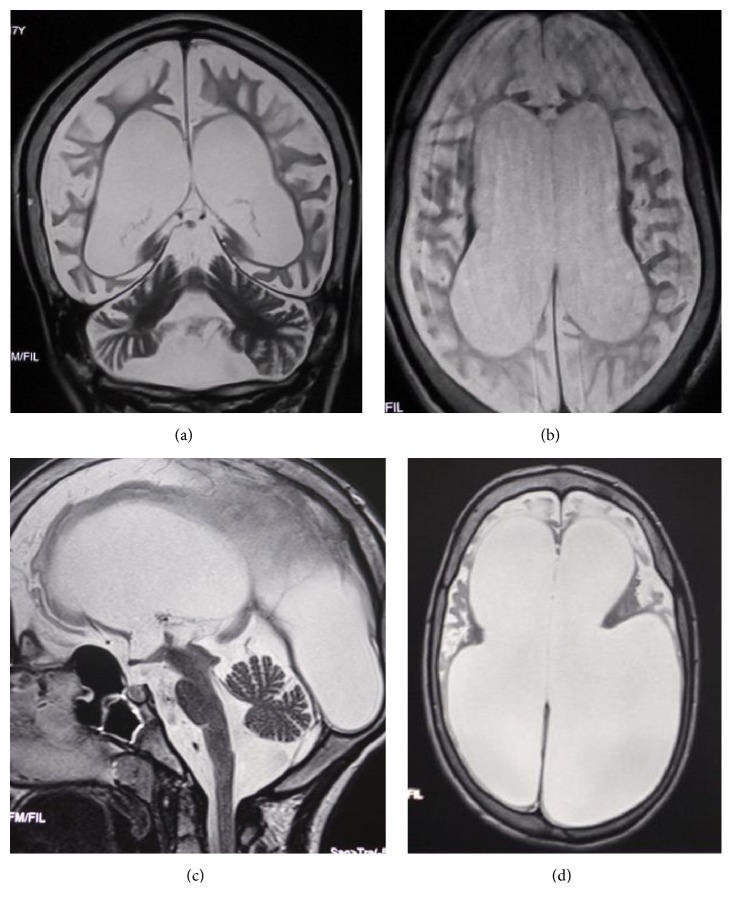
Brain MRI of the patients V.1 ((a) and (b)) and V.2 ((c) and (d)). (a) Coronal T2 weighted image showing enlargement of the cortical furrows and the ventricular cavities related to a diffuse cortical and subcortical atrophy. (b) Axial T2 weighted sequence showing the huge cortical and subcortical atrophy. (c) T2 weighted sequence shows dilatation in all ventricular cavities as a result to a diffuse and severe atrophy in the brainstem and the cerebellar and the sustentorial area. (d) T2 weighted sequence showing a major ventricular dilatation due to the severe cortical atrophy.

The brain-MRI revealed a diffuse brain atrophy especially giving it a laminated appearance (Figures 2(a) and 2(b)). The EEG revealed a symmetrical background activity desynchronized and microvoltated without any epileptic sings. The ENMG was normal. Otherwise, the cardiac ultrasonography revealed aortic insufficiency with an aortic valve prolapse without any aspect of cardiomyopathy.

Patients V.2 and V.3: the other sister was 14 years old; she is the youngest coming from a twin pregnancy and was admitted in pediatrics department to assess a progressive mental regression. The story dates back to the age of 6 when she had a feverish episode at 43 degrees treated with antipyretics. Then the occurrence of walking disorders with frequent falls due to the total loss of muscular tone results in repeated cranial trauma. Subsequently she began to present generalized tonic-clonic epileptic seizures and progressive psychomotor deterioration.

The neurological examination found a patient in good general condition; the segmental muscle strength was preserved, with a pyramidal spasticity in the four limbs, bilateral positive Babinski sign, with normal cranial nerves examination. There were some orthopedic deformities. Both feet were clubfeet in equine varus and the metacarpophalangeal joints of both hands were deformed with stiffness at flexion. The thorax was asymmetrical but there was no deformity in the spine.

Brain-MRI showed an important ventricular dilatation due to the diffuse homogenous atrophy of the sustentorial structures and the brainstem (Figures 2(c) and 2(d)). The EEG revealed normal findings. Chromatography of amino acids was normal. The abdominal ultrasound was normal. The other twin sister presented the same clinical evolution and symptoms.

### 3.2. Linkage Analysis

We genotyped 2.6 million markers in the DNA samples of the 3 patients and both parents. No pathogenic CNV was identified within all the genomes of the three patients and therefore we searched for regions of homozygosity (ROH). This analysis revealed several ROH with more than 500 markers and 2Mb in length. Comparison between patients found that the twin sisters V.2 and V.3 have the same genotype and have in common with the patient V.1 only three ROH at chromosomes 3, 13, and 15 containing 2529, 2084, and 2392 markers, respectively.

**Figure 3:**
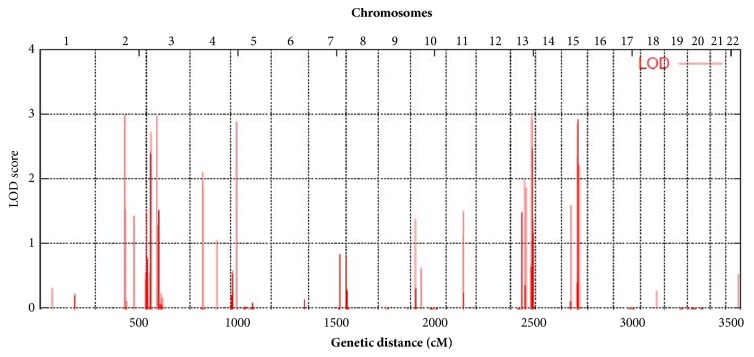
Plot of whole genome linkage analysis. Parametric multipoint LOD scores indicate linkage to three loci on 3p24.1-22.2, 13q33.2-34, and 15q22.2-25.1 with a maximum LOD score close to three.

Whole genome LOD score analysis under the assumption of full penetrance and a disease allele frequency of 0.001 revealed a significant LOD score over 2.9 on chromosomes 3p24.1-22.2, 13q33.2-34, and 15q22.2-25.1 (Figure 3). These three linked regions of 8.4Mb, 5.4Mb, and 9.9Mb in length contain 34, 19, and 252 positional candidate genes, respectively. Two significant LOD scores have also been obtained for chromosomes 2 and 5, but these loci were less than 500 markers and did not contain any gene.

Sanger sequencing of positional candidate genes in relation to the aetiology of the disease, particularly hydrocephalus and epileptic encephalopathy (*CCDC33*,* BBS4*, and* CSNK1G1* genes), found no pathogenic mutation compatible with autosomal recessive inheritance.

### 3.3. Exome Sequencing

**Figure 4:**
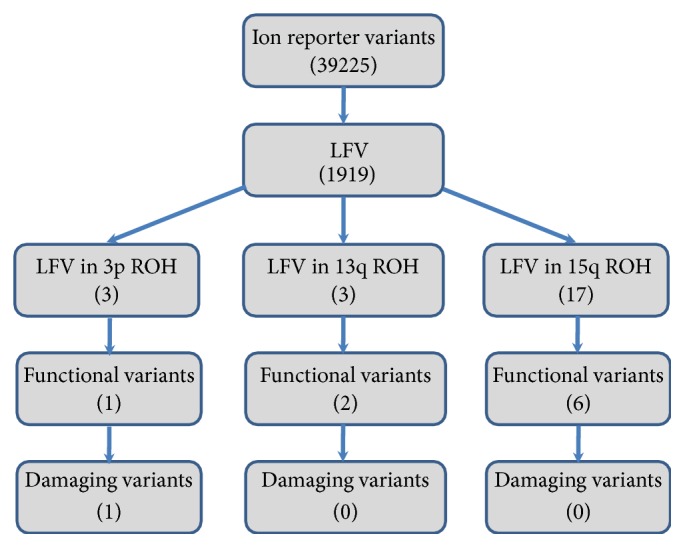
Pipeline for filtration of the damaging variants.

**Figure 5:**
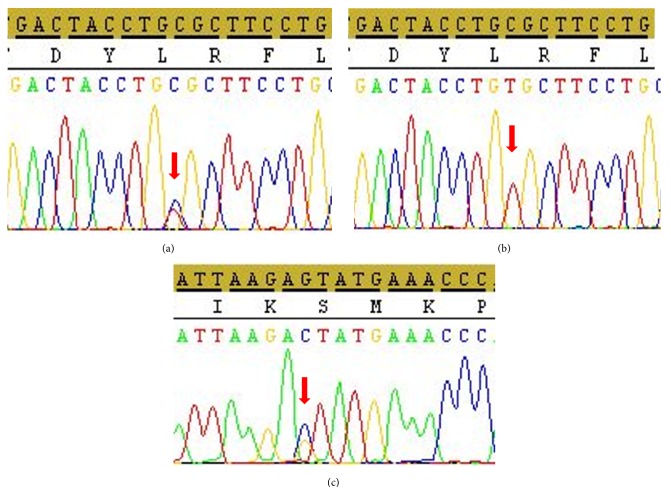
Sanger sequencing plot of* GLB1 *showing the p.Arg201Cys mutation at heterozygous state in the father IV.1, the mother IV.2, and her sister IV.3 (a) and at homozygous state in patients V.1, V.2, and V.3 (b).* EXOSC8* p.Ser272Thr mutation at heterozygous state found in patients V.1, V.2, and V.3, theirs mother IV.2, and their aunt IV.3 (c). Arrows indicate mutation positions.

Since there were 305 candidate genes in the three ROH linked regions, we opted for whole exome sequencing of DNA from patients V.3 and his father IV.1. The primary filtering by Ion Reporter software led to the identification of 39,225 variants consisting of SNVs, MNVs, and INDELs in 13,296 genes. These mutations were subsequently filtered according to the criterion of the frequency of the minor allele less than 5% that yielded 1919 low frequency variants (LFV, MAF < 5%) in 1826 genes (Figure 4). The coverage obtained for these variants ranged from 25 to 400 with more than 85% being over 50. Considering only the ROH linked regions determined by linkage analysis, the patient presented 3, 3, and 17 LFV at the 3p24.1-22.2, 13q33.2-34, and 15q22.2-25.1 loci, respectively, in 20 genes. Among them, we found 1, 2, and 6 functional variants which were all missense mutations (Table 1). Only the p.Arg201Cys missense mutation in* GLB1* was reported to be damaging and responsible for the gangliosidosis type II disease. The WES data showed that the father IV.1 was heterozygous for all these variants. The p.Arg201Cys was validated by Sanger sequencing in the patient and all the family members showing a cosegregation of the mutation with the disease. The three patients were homozygous and the parents were heterozygous for the mutation (Figures 5(a) and 5(b)). We did not observe pathogenic homozygous variants outside the ROH linked regions in other genes in relationship with the clinical phenotype. However, we found the* EXOSC8* p.Ser272Thr mutation at heterozygous state in the patient V.1 but not in his father. Sanger sequencing found this mutation at heterozygous state in all the patients, their mother, and their aunt (Figure 5(c)).

## 4. Discussion

Even if the age of onset of the first clinical signs was 6 years, the evolution and the clinical presentation fit the juvenile form and could be classified as GM1-type II. The disease classification in the literature is found to be different from a report to another. Besides the classical definition described previously, some authors define type I when the signs appear before 3 years of age [16] and type II into two subclasses: the type II late infantile form where the signs might appear under 3 years of age and the juvenile form where symptoms start between 3 and 5 years of age as described by Wolfe et al. [17].

Our patients had some specific GM1 clinical features such as mental retardation, refractory seizures, and skeletal abnormalities. However, they did not present the pathognomonic features of the disease, which made the diagnosis not obvious. The particularity of this observation is the major brain atrophy present in the three cases, with an aspect of laminated cortex. This aspect did contribute to the delay of the diagnosis due to the immense ventricular dilatation and the brain atrophy, which is not commonly reported in this disease.

Usually, the clinical suspicion of the GM1-gangliosidosis is based on the existence of signs indicating the massive storage of the GM1 ganglioside in the organs. Those signs are diverse: coarse facial features, corneal clouding, opacification of the cornea, cherry-red macula, gingival hypertrophy, hepatosplenomegaly, skeletal dysostosis, vacuolated lymphocytes, and the psychomotor regression [5, 8]. The diagnosis could be more difficult if these signs are missing.

In their study, Bidchol et al. [8] analyzed clinical information of 46 families and confirmed that the developmental delay was the most common clinical presentation while seizures were less frequent, observed only in 24% of cases. Our patients had generalized refractory seizures complicating feverish episodes. In the reported cases of the literature, seizures were described shortly and the common character was the refraction to antiepileptic drugs [7].

Patients with GM1 type II (juvenile form) start a normal neurological development until their late childhood [7]. This particularity gives the patients the chance to be treated with enzyme replacement therapy, bone marrow transplantation, and even cell therapy, if the diagnosis is made in early stage. Unfortunately, for our patients, the diagnosis came late and the access to those therapies is difficult in our structures.

Few articles described the neuroimaging findings in patients with GM1 gangliosidosis type 1 and type 3. To our knowledge, the MRI aspects in type II were not wildly described. The abnormalities commonly seen in the GM1-gangliosidosis are signal hypointensities in the grey nuclei and a delay in white matter myelination [6, 18, 19]. The MRI in the three patients showed a homogenous atrophy of the whole brain structures. This aspect may be due to the severe and repetitive brain suffering resulting in significant neuronal loss.

The disease in this studied family had some clinical and radiological atypia that delayed diagnosis and better management. Today, advances in high-throughput sequencing technology have been fundamentally transforming our understanding of genetics, particularly in the case of syndromic diseases for many reasons. First, the clinical pictures traditionally described for the various syndromes are often partly present and therefore clinically hardly recognizable. In addition, some clinical pictures can be caused by mutations on different genes, at the same time; mutations in a gene can lead to various clinical pictures. Even in the case of classical Mendelian diseases, there is thus no strict correlation between the responsible genes and the clinical pictures. These difficulties are clearly illustrated in the lysosomal diseases. Exome sequencing enables sequencing all human genes in a single analysis and is the most cost-effective method in the presence of suspicion of lysosomal storage disease and generally for most metabolic and syndromic diseases.

The interpretation of sequencing data, however, remains a challenge because of the great variability in the sequence of our genome and the high frequency of benign changes, and results may take months to report. The difficulty then resides in the distinction between the pathogenic variant at the origin of the disease and the benign variants. In order to reduce the number of variants to be analyzed and the time to the disease diagnosis, the NGS analysis could be preceded by chromosomal microarray genotyping that leads to determining the gene locus.

The combination of CMA and NGS technologies allowed the genetic diagnosis very quickly in this family. Indeed, the use of CytoScan DNA chips allowed first the identification of three significant loci at 3p24.1-22.2, 13q33.2-34, and 15q22.2-25.1 chromosomal regions. Analysis of the NGS data then focused only on these chromosomal regions, which reduced the number of variants from 39225 to 23. Among them, there were nine functional mutations, and only the p.R201C in* GLB1* was known to be damaging, confirming the diagnosis of the GM1-gangliosidosis in the studied family. The p.R201C mutation is very common and was particularly reported in the juvenile form of GM1-gangliosidosis [7, 20–22]. Another amino acid substitution affecting the same codon (p.R201H) has been identified in both GM1 and MBD patients [23–25].

However, since the three patients had some deviations from the common GM1 clinical phenotype, particularly the severe and diffuse brain atrophy, NGS data analysis was extended outside the chromosomal linked regions and found another pathogenic mutation, the p.Ser272Thr in* EXOSC8*. This mutation, found at heterozygous state in all the patients, their mother, and their aunt, was reported to affect mRNA metabolism and cause the autosomal recessive pontocerebellar hypoplasia type 1C [26]. They reported that MRI of patients with homozygous Ser272Thr mutation showed vermis hypoplasia, immature myelination, cortical atrophy, and thin corpus callosum. Moreover, the MRI of patients with mutations in another exosome compound EXOSC9 showed severe cerebellar and moderate cerebral and brainstem atrophy [27]. Taking into account these results, the p.Ser272Thr in* EXOSC8 *mutation could act as heterozygous-modifying factor that exacerbates the brain atrophy in the clinical presentation of our GM1 patients with homozygous* GLB1* mutation.

## 5. Conclusion

We describe a family consisting of three patients with juvenile-onset GM1-galactosidosis whose diagnosis took a long time as this pathology represents an overlapped disease spectrum. The genetic diagnosis was possible by the combination of WES and linkage analysis using CMA, which significantly reduces the number of genetic variants to be analyzed. We identified the first* GLB1* mutation in Morocco and North Africa, the p.R201C, in patients with unusual phenotype extending the clinical spectrum of the GM1-gangliosidosis. Our study highlights the importance of WES in assessing patients with undiagnosed diseases and generally all neurogenetic diseases with a high clinical heterogeneity.

## Figures and Tables

**Table 1:** List of low frequency functional variants in the three linked ROH regions.

Locus	Gene name	cDNA nucleotide change	AA change	rsnumber	MAF	Clinvar
3p24.1-22.2	GLB1	c.601C>T	p.Arg201Cys	rs72555360	0.0002	Damaging
13q33.2-34	MYO16	c.4199C>T	p.Ala1400Val	rs7995379	0.0060	Benign
	ING1	c.374T>G	p.Leu125Arg	rs199777754	0.0010	Benign
15q22.2-25.1	FBXL22	c.132G>C	p.Glu44Asp	rs142301663	0.0030	Benign
	LCTL	c.155C>T	p.Thr52Met	rs141474589	0.0030	Benign
	IQCH	c.2794G>C	p.Val932Leu	rs3985641	0.0020	Benign
	C15orf39	c.2834G>A	p.Gly945Asp	rs3743211	0.0500	Benign
	CHRNA5	c.488C>T	p.Pro163Leu	rs55863434	0.0003	Benign
	FAH	c.565G>A	p.Val189Ile	rs145389125	0.0020	Benign

## Data Availability

The data used to support the findings of this study are included within the article.
